# Strange case of a suspected foreign body after cephalic pancreatoduodenectomy

**DOI:** 10.1055/a-2408-9723

**Published:** 2024-09-25

**Authors:** Pablo López-Guillén, Carolina Mangas-Sanjuan, Juan Martínez-Sempere

**Affiliations:** 1 Gastroenterology, General University Hospital of Alicante, Alicante, Spain

A 58-year-old man underwent cephalic pancreatoduodenectomy for pancreatic adenocarcinoma. After 1 year of surgery, he experienced a transient episode of diffuse abdominal pain with nausea and vomiting. As a result, contrast-enhanced abdominal computed tomography scan was performed, which revealed partial absence of the wall of the jejunal afferent loop, suggestive of necrosis ( Fig. 1 ).

**Fig. 1:**
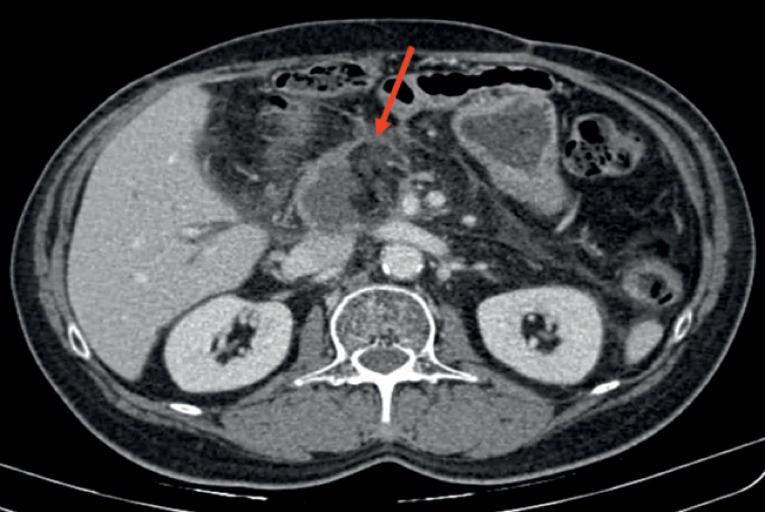
Axial section of contrast-enhanced abdominal computed tomography scan revealed partial absence of the wall of the jejunum loop used for anastomosis with the biliary system (red arrow), suggesting necrosis of this loop.

We performed diagnostic gastroscopy, which revealed a yellow jelly-like suspected foreign body inside the afferent jejunal loop ( Video 1 , Fig. 2 ). We failed to remove the suspected foreign body using foreign body forceps. Biopsy samples showed the presence of amorphous material and inflammatory cells. After 1 week, the foreign body was removed endoscopically ( Video 1 , Fig. 3 ).

**Fig. 2:**
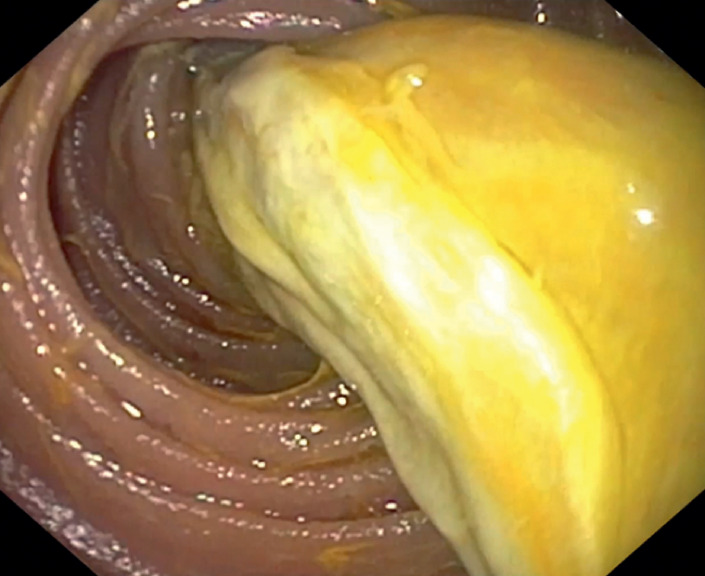
Initial gastroscopy showed a yellow jelly-like “foreign body” inside the afferent jejunal loop.

**Fig. 3:**
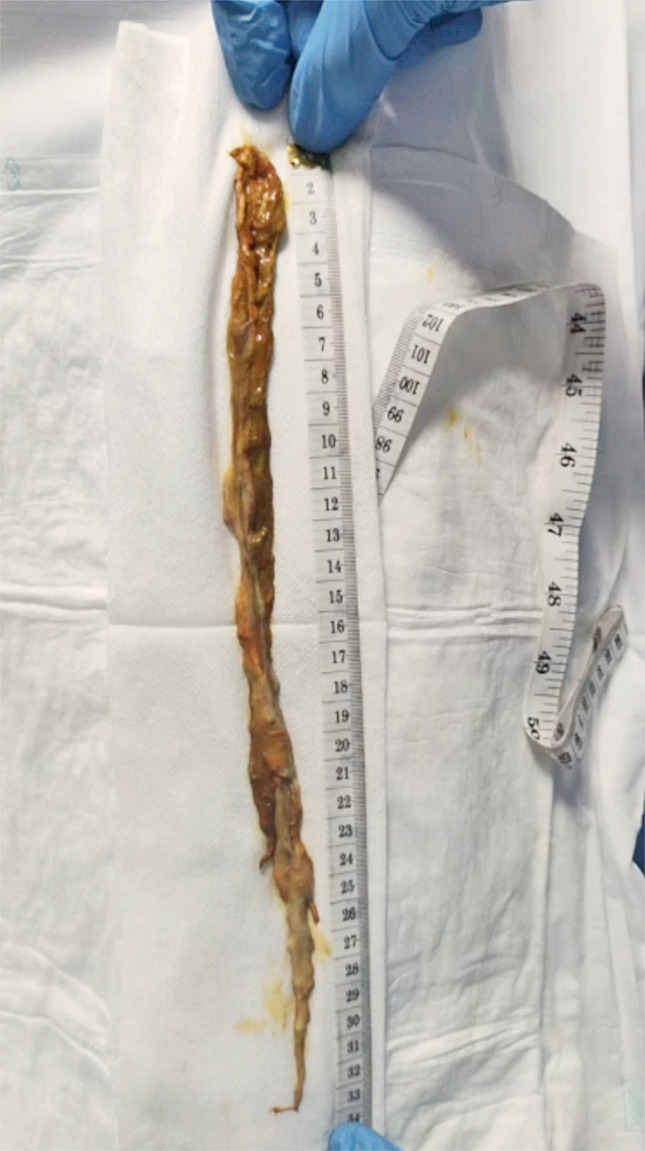
Macroscopic view of the 33-cm-long suspected foreign body.

Initial gastroscopy revealed a suspected foreign body inside the afferent jejunal loop in a 58-year-old man who underwent cephalic pancreatoduodenectomy for pancreatic adenocarcinoma. A second gastroscopy was performed to extract the suspected foreign body.Video 1

Pathological analysis of the tissue revealed low levels of inflammatory infiltrates. The presence of the muscularis propria layer in the tissue confirmed that the suspected foreign body was a fragment of the jejunal wall with abundant necrohemorrhagic debris ( Fig. 4 ).

**Fig. 4:**
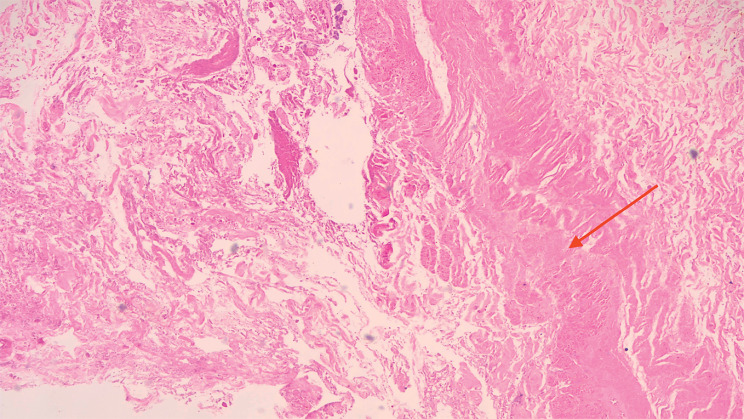
Hematoxylin and eosin-stained axial section of the suspected foreign body revealed presence of the muscularis propria layer (red arrow).

Cephalic pancreatoduodenectomy (Whipple procedure) is a complex surgery performed in cases of malignant tumors of the pancreatic head, ampulla, and distal bile duct or, less frequently, for chronic pancreatitis 1 . Complications occur in at least 30% of these patients 1 2 . The most frequent complications are wound infections, intraabdominal collections, delayed gastric emptying, anastomotic leak, and postoperative hemorrhage 1 2 . Less common complications include gastrojejunal ulceration, afferent loop syndrome, and ischemia 3 . However, the development of a suspected foreign body from the jejunal wall has never been reported. Its etiology remains unclear, although an ischemic or infectious mechanism is suspected.

In conclusion, the formation of a suspected foreign body inside the afferent jejunal loop after cephalic pancreatoduodenectomy is a rare complication. Abnormal radiological findings in the afferent jejunal loop may indicate the presence of this complication.

Endoscopy_UCTN_Code_CCL_1AC_2AH

## Notes

Endoscopy E-Videos https://eref.thieme.de/e-videos
*E-Videos* is an open access online section of the journal *Endoscopy* , reporting on interesting cases and new techniques in gastroenterological endoscopy. All papers include a high-quality video and are published with a Creative Commons CC-BY license. Endoscopy E-Videos qualify for HINARI discounts and waivers and eligibility is automatically checked during the submission process. We grant 100% waivers to articles whose corresponding authors are based in Group A countries and 50% waivers to those who are based in Group B countries as classified by Research4Life (see: https://www.research4life.org/access/eligibility/ ). This section has its own submission website at https://mc.manuscriptcentral.com/e-videos .
